# Association of TLR5 Gene Polymorphisms in Ulcerative Colitis Patients of North India and Their Role in Cytokine Homeostasis

**DOI:** 10.1371/journal.pone.0120697

**Published:** 2015-03-19

**Authors:** Naresh Kumar Meena, Vineet Ahuja, Kusumlata Meena, Jaishree Paul

**Affiliations:** 1 School of Life Sciences, Jawaharlal Nehru University, New Delhi, India; 2 Department of Gastroenterology, All India Institute of Medical Sciences, New Delhi, India; 3 Department of Obstetrics and Gynaecology, SMS Medical College, Jaipur, India; University of Thessaly, Faculty of Medicine, GREECE

## Abstract

**Background and Aim:**

In health, TLR signaling protects the intestinal epithelial barrier and in disease, aberrant TLR signaling stimulates diverse inflammatory responses. Association of TLR polymorphisms is ethnicity dependent but how they impact the complex pathogenesis of IBD is not clearly defined. So we propose to study the status of polymorphisms in TLR family of genes and their effect on cytokines level in UC patients.

**Methods:**

The genotypes of the six loci TLR1-R80T, TLR2-R753Q, TLR3-S258G, TLR5-R392X, TLR5-N592S and TLR6-S249P were determined in 350 controls and 328 UC patients by PCR-RFLP and sequencing. Cytokine levels were measured by ELISA in blood plasma samples. Data were analyzed statistically by SPSS software.

**Results:**

TLR5 variants R392X and N592S showed significant association (p = 0.007, 0.021) with UC patients but TLR 1, 2, 3, 6 variants did not show any association. Unlike other studies carried out in different ethnic groups, TLR 6 (S249P) SNP was universally present in our population irrespective of disease. Genotype-phenotype correlation analysis revealed that the patients having combination of multiple SNPs both in TLR5 and TLR4 gene suffered from severe disease condition and diagnosed at an early age. The level of TNFα (p = 0.004), IL-6 (p = 0.0001) and IFNγ (p = 0.006) significantly increased in patients as compared to controls having wild genotypes for the studied SNPs. However, there was decreased level of TNFα (p = 0.014), IL-6 (p = 0.028) and IFNγ (p = 0.001) in patients carrying TLR5-R392X variant as compared to wild type patients. Patients carrying two simultaneous SNPs D299G in TLR4 gene and N592S in TLR5 gene showed significant decrease in the levels of TNFα (p = 0.011) and IFNγ (p = 0.016).

**Conclusion:**

Polymorphisms in TLR 5 genes were significantly associated with the UC in North Indian population. The cytokine level was significantly modulated in patients with different genotypes of TLR4 and TLR5 SNPs.

## Introduction

Crohn’s disease (CD) and ulcerative colitis (UC) are together referred to as inflammatory bowel disease (IBD). IBD is represented as chronic remittent inflammatory conditions of the gastrointestinal tract. Exact etiology of IBD is unknown but according to the current hypothesis it is believed that during IBD, there is an aberrant immune response in genetically susceptible individual against bacterial flora of the intestine [1–3].

Recognition of antigens have key role in innate mucosal immunity. Various cell surface receptors like toll-like receptors (TLR) recognize different microbial-associated molecular patterns (MAMPS), not expressed by the host but shared by many microbes [4]. TLRs are a family of trans-membrane proteins that act as microbial pattern recognition receptors. TLRs are differentially expressed either constitutively or induced by many distinct cell types throughout the whole gastrointestinal tract [5]. They are crucial initiators of innate immune responses. There are currently 11 known mammalian TLRs. They are transmembrane receptors that are found either on the cell membrane (TLR1, 2, 4, 5 and 9) or on intracellular organelles (TLR3, 7 and 8) [6]. Individual TLRs differentially activate distinct signaling events via diverse cofactors and adaptor proteins mediating specific immune responses.

TLR5 is highly expressed in colonic epithelial cells (CECs). The TLR5 ligand, flagellin can modulate the balance between T regulatory and T effecter cells in IBD [7]. Other TLRs can also affect cytokine homeostasis, T cell maturation and proliferation through their respective ligands. TLR genes are also subject to single nucleotide polymorphisms (SNPs) leading to an aberrant immune response during disease conditions. Worldwide studies show association of TLR polymorphisms with various diseases including IBD. According to various studies; mutations in TLR genes may either enhance or suppress intestinal inflammation. A TLR5-stop polymorphism (R392X) in which a point mutation at nucleotide position 1174, generates a stop codon rendering TLR5 inactive [8]. It is a relatively common polymorphism with a 5% allele frequency. However, the frequency of TLR5-stop SNP was significantly lower in CD patients as compared to unaffected relatives and unrelated controls [9]. The TLR2 R753Q and TLR1 R80T SNPs were found to be associated with pancolitis in UC [10]. A negative association was observed-between TLR6 S249P SNP and proctitis in UC patients [11]. TLR3 expression was significantly downregulated in CD patients, both in inflamed and non-inflamed tissue [11]. SNPs in TLR4 gene has been shown to be associated with *H*. *pylori* infection and resulted in a modified pattern of inflammatory cytokines and chemokines in the gastric mucosa [12]. SNPs in TLR genes may influence the expression of inflammatory cytokines and are probably associated with susceptibility to ulcerative colitis. No association analysis has been carried out so far between SNPs and pro-inflammatory cytokine levels during UC. It has also been speculated that mutations in a single TLR are insufficient to explain the complex pathogenesis of IBD. So we propose to study the status of polymorphisms present in single or in multiple TLR genes of North Indian population and their role in the cytokine homeostasis with respect to different combinations of SNP. We have included three important circulating pro-inflammatory cytokines TNFα, IL-6 and IFN-γ for our study.

## Materials and Methods

### Study Subjects

Study was conducted on 350 controls and 328 UC patients of North Indian population, UC patients and controls (patients without gastrointestinal and liver disease) were recruited through the department of Gastroenterology of AIIMS, New Delhi, India. Control samples were also collected from, SMS Medical College, Jaipur, India. Samples were matched for age and sex. Diagnosis was done through endoscopic, histopathological and radiological examinations by following ECCO (European Crohn’s and Colitis Organization) guidelines. Montreal classification was followed for phenotypic analysis of UC patients (Table 1) [13].

**Table 1:** Demographic and clinical features of UC and Controls.

	UC (n = 328)	Controls (n = 350)
Sex (M/F)	207/121	203/147
Duration of disease, mean (years)	4.89 (0.2–31)	-
Age at diagnosis mean ± SD (years)	30.6±09.6 (10–65)	32.2±11.9 (18–61)
15–40	202 (61.58%)	214 (61.14%)
>40	126 (38.41%)	136 (38.85%)
Disease behavior, n (%)		
Remission	156 (47.56%)	-
Active	172 (52.43%)	-
Disease extent, n (%)		
Rectum	122 (37.19%)	-
Left sided	70 (21.34%)	-
Pancolitis	136 (41.46%)	-
Food type (%)		
Vegetarian	198 (60.36%)	203 (58.00%)
Non vegetarian	130 (39.63%)	147 (42.00%)
Social status (%)		
Rural	148 (45.12%)	227 (64.85%)
Urban	180 (54.87%)	123 (35.14%)

M = Male, F = Female, n = Number

### Ethics Statement

Ethical approval for the study was obtained from Institute Ethics Committee, All India Institute of Medical Sciences, Room No. 102, 1^st^ Floor old O.T. block, Ansari Nagar, New Delhi, India (Ref. No.: IEC/NP-161/2010). Informed written consent was obtained from all patients and controls. At the time of sample collection subject details were collected according to the designed protocols for the study.

### Blood Plasma and Genomic DNA Isolation

Blood sample was collected in vacutainer (*BD*, *NJ*, *USA*) containing anticoagulant K_2_EDTA solution by using venipuncture in vein. Within 2 hr of blood collection, sample was centrifuged at 1500 g for 15 minute at 4°C for plasma separation [14]. Plasma samples were stored at −80°C immediately. Total genomic DNA was isolated from peripheral blood leucocytes following standard protocols [15].

### Primer Designing and Validation

Gene sequences of the TLR family available in national centre for biotechnology information database (Genbank) were used to design primer sets. Primer express software v3.0 (Applied Biosystems, California, USA) was used to check hairpin loop and formation of dimer in designed primer sets. BLAST (Genbank program) analysis was used to confirm if the primers were complimentary to only target genes. Designed primer set sequence, gene names, SNP names, PCR product sizes and corresponding restriction enzymes used for RFLP are shown in Table 2. All the primers were commercially synthesized from Sigma-Aldrich Corporation (Bangalore, India).

**Table 2:** Detail of PCR primers used for Restriction fragment length polymorphism.

Gene	SNP name	alleles	Forward/Reverse primer sequence (5’-3’)	PCR product size	Restriction enzyme
TLR1	R80T	G>C	F-5’ TTCCTAAAGACCTATCCCAG3’	341 bp	Hinf1
			R-5’GAGCAATTGGCAGCACACTAG3’		
TLR2	R753Q	G>A	F-5’ CATAAGCGGGACTTCATTCCTGCAA3’	372 bp	Pst1
			R-5’GATCCCAACTAGACAAGACTGGTCT3’		
TLR3	S258G	A>G	F-5’GGGTCCCAGCCTTACAGAGA3’	341 bp	BsuRI
			R-5’CAATCTTGGGGAGTGAGGCA3’		
TLR5	R392X	C>T	F-5’GGTAGCCTACATTGATTTGC3’	346 bp	Dde1
			F-5’GGATTCTCTGAAGGGGTTTAT3’		
TLR5	N592S	A>G	F-5’GACTAAGCCTCAACTCCAACA3’	314 bp	Mun1
			R-5’GACTTCCTCTTCATCACAACC3;		
TLR6	S249P	T>C	F-5’CTAGTTTATTCGCTATCCAAG3’	312 bp	Eco47I
			R-5’TTGTCAATGCTTTCAATGTCG3’		

PCR = Polymerase chain reaction, TLR = Toll like receptor, bp = Base pairs, SNP = Single nucleotide polymorphism.

### Polymerase Chain Reaction (PCR)

PCR reaction was done in 0.2 ml PCR tubes by using ABI PCR machine. A typical PCR reaction (20 μl) included 2 μl of 10X PCR buffer (containing 750mM Tris-HCl (pH 8.8 at 25^0^ C), 200mM (NH_4_)_2_ SO_4,_ 0.1% Tween-20), 2 μl of dNTP mix (containing 2mM of each dNTP), 1.6 μl MgCl _2_ (final concentration 2mM), 0.8 μl (20 pmol) of each primer forward as well as reverse and 0.2 μl of Taq DNA polymerase (5U/μl (MBI Fermentas, USA), 11.1 μl of autoclaved nuclease free water and 1.5 μl of template DNA (50ng). The PCR conditions were as follows; one cycle of initial denaturation at 94^0^ C for 5 min, then 30 cycles of 94^0^ C for 15–30s, annealing temperature was decided depending on Tm value of primers sets. Annealing was carried out for 40s to1min., followed by extension at 72^0^ C for 40s to 1 min and last cycle of final extension was carried out at 72^0^ C for 5 to 8 min. (depending on the product size) and finally cooled down to 4^0^ C. The products were analyzed by electrophoresis on a 1.2% agarose gel at 5V/cm for an appropriate time period.

### Restriction Fragment Length Polymorphism (RFLP)

PCR product was digested overnight with suitable restriction enzyme (Fermentas and NEB). Digestion temperature and buffers were used as per manufacturer's protocol. After digestion, electrophoresis was performed with 1X gel loading buffer (GLB) containing: 5% Ficoll type-400, 10 mM EDTA at pH 8.0 and 0.04% xylene cyanol. Agarose gel concentration (1–2.4%) and time of electrophoresis were set according to PCR product sizes.

### Sequencing

Three samples in each category of SNP scored by the RFLP analysis were analyzed by sequencing along with their respective controls. Amplified PCR product was cloned in pGEMT vector. By using ABI big Dye Terminator cycle sequencing kit v1.1 (Applied Biosystems) sequencing reaction was performed in ABI Prism 310 Genetic Analyzer (Applied Biosystems). BioEdit sequence alignment editor software was used for aligning two sequences (allow ends to slide) using pair wise alignment.

### Enzyme Linked Immunosorbent Assay (ELISA)

TNFα, IL-6 and IFN-γ in human blood plasma was measured by ELISA (ebiosciences, CA, USA). Minimum detectable level of ELISA kit was 4pg/ml for TNFα and IFN-γ, 2pg/ml for IL-6. An additional standard consisting of IFN-γ, TNFα and IL-6 recombination proteins were used as a standard to measure any differences between ELISA assay performances. The assays were performed according to the manufacturer’s instructions. The optical density was read using micro plate reader at λ = 450 nm. Results represented as pg/ml are mean of 4 to 8 samples in each category.

### Statistical Analysis

Cases and controls were analyzed statistically using SPSS software version 17. Allelic frequencies were tested for Hardy-Weinberg equilibrium. Observed genotypes were analyzed using various tests such as, Pearson Chi Square, Fisher’s exact test, Student’s t test in both controls and patient samples. Genotype-phenotype association analyses were performed by using allele frequency. The significance level of P ≤0.05 was chosen for all sets.

## Results

Single nucleotide polymorphisms were analyzed in TLR genes by using PCR-RFLP method. Sequencing was done to further confirm RFLP data and to detect nucleotide change. D299G and T399I SNPs of TLR4 gene reported earlier for 199 individuals [16] was also included in this study to select out patients having simultaneous mutations both in TLR4 and TLR5 genes. This was used to assess genotype-phonotype correlation and ELISA analysis.

### Association of TLR5 SNPs

**Fig 1:**
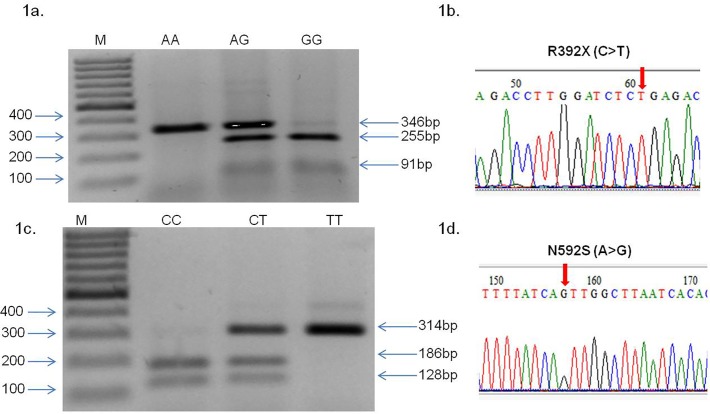
RFLP profiling of TLR5 R392X (C>T), N592S (A>G) and sequencing chromatogram. (1a) 346 bp DNA fragment was amplified by PCR, incubated with Dde1 restriction endonuclease for 12–16 hour, R392X (C>T) genotypes were deduced from migration profile on a 2% agarose gel electrophoresis, lane M showing 100bp molecular marker, wild type DNA visible in lane CC with band size 346bp, heterozygous mutant is visible in lane CT with three bands 346+255+91 bp, homozygous mutant (TT) is visible with 255+91 bp band. (1b-1d) Sequencing chromatogram results of TLR5 (1b) R392X (C>T) and (1d) N592S (A>G) SNP. Arrows show position of nucleotide change. (1c) 314 bp DNA fragment was amplified by PCR, incubation with MunI restriction endonuclease for 12–16 hr., N592S A>G genotypes were deduced from migration profile on a 2% agarose gel electrophoresis, lane M showing 100 bp molecular marker, wild type DNA in lane AA with band size 186+128 bp, heterozygous mutant in lane AG with three bands 314 bp +186 bp+128 bp, homozygous mutant with single 314bp band.

Fig. 1 (A-D) shows representative results of RFLP and sequencing carried out with TLR5 gene variants (R392X and N592S). Detailed analysis showing the location of SNPs, genotyping and restriction digestion is shown in S1 Fig. Association of R392X and N592S SNPs of TLR5 with UC patients and controls are represented in Table 3. Allele frequency of R392X were significantly different as compared to 1.7% in controls versus 4.4% in patients (odds ratio 2.535, confidence interval 1.262–5.092, p value = 0.007). In case of N592S SNP, frequency was also significantly different as compared 11% in controls versus 9% in patients (odds ratio = 0.633, confidence interval 0.429–0.934 and p value = 0.021). Statistical power of the study was calculated 99.99% for R392X and 71.17% for N592S SNP. The association of SNP with the disease revealed that while R392X was positively correlated whereas N592S was negatively correlated with the disease activity as compared to controls. Allele frequencies were in Hardy-Weinberg equilibrium in all patients and in the control groups for these SNPs.

**Table 3:** Genotype and allele distribution for TLR variants in UC patients and controls.

SNPs (Gene)	Total	Wild type	Het. Mutant	Homo. Mutant	MAF	Odds ratio (CI)	p value
**R392X (TLR5)**							
Controls	350	338 (96.5%)	12 (3.42%)	0 (0.0%)	0.017		
UC	328	300 (91.46%)	27 (8.23%)	1 (0.30%)	0.044	2.535 (1.262–5.092)	0.007*
**N592S (TLR5)**							
Controls	350	269 (76.85%)	80 (22.85%)	1 (0.28%)	0.11		
UC	328	271 (82.62%)	51 (15.54%)	6 (1.82%)	0.09	0.633 (0.429–0.934)	0.021*
**S249P (TLR6)**							
Controls	350	0 (0.0%)	0 (0.0%)	350 (100%)			
UC	328	0 (0.0%)	2 (0.60%)	326 (99.3%)			
**R753Q (TLR2)**							
Controls	350	350 (100%)	0 (0.0%)	0 (0.0%)			
UC	328	327 (99.69%)	1 (0.30%)	0 (0.0%)			
**R80T (TLR1)**							
Controls	350	350 (100%)	0 (0.0%)	0 (0.0%)			
UC	328	328 (100%)	0 (0.0%)	0 (0.0%)			
**S258G (TLR3)**							
Controls	350	350 (100%)	0 (0.0%)	0 (0.0%)			
UC	328	328 (100%)	0 (0.0%)	0 (0.0%)			

Het = Heterozygous, Homo = Homozygous, CI = Confidence interval, MAF = Minor allele frequency

* showing significant p value ≤ 0.05.

### Association of TLR1, TLR2, TLR3 and TLR6 SNPs

While analyzing samples for polymorphism in TLR2 gene, we detected only one heterozygous mutant in a patient sample for the SNP R753Q when we digested with PstI restriction enzyme and the rests were all wild type for both patients and controls (Table 3). Next, polymorphism in TLR6 gene was scored for S249P SNP. Out of 328 patients, interestingly, we observed 326 homozygous mutants and only 2 heterozygous mutants for this SNP. Further we confirmed that in our population, even control samples were all homozygous mutant for this SNP. We did not observe any mutant sample (heterozygous or homozygous) for R80T SNP in TLR1 and S258G SNP in TLR3 gene (Table 3).

### Genotype—Phenotype Analysis

Minor and major allele frequencies were stratified according to phenotypic subgroups as observed in different SNP combinations (Table 4). Patients having multiple mutations in the TLR4 gene, referred here as (D299G/T399I combination) were subjected to genotype-phenotype analyses for sex, age at diagnosis, disease behavior, disease extent, food habit and social status (Table 4). No association with the disease was observed with respect to sex, food type and social status of patients. However, we observed higher (7%) minor allele frequency in patients with younger age group (15–40 years) as compared to the older age group (>40 years, 3%) in D299G/T399I SNPs combination (Table 4). Positive association was observed where SNPs D299G and T399I were present in combination with pancolitis and left-sided colon patients (8%) compared to patients where rectum area (2%) was involved. When we analyzed phenotypes of co-existing SNPs D299G (TLR4) and N592S (TLR5), we observed that only age factor at diagnosis shows association with minor allele frequency. Patients with younger age group show 4% frequency of minor allele as compared to 0% in patients of older age group (Table 4). We conducted another SNP interaction study, (R392X/N592S) however sample size carrying two simultaneous mutations in TLR5 gene was found to be small for genotype phenotype analysis.

**Table 4:** Genotype and allele frequencies of D299G/T399I and D299G/N592S combinations in UC cases stratified by phenotype.

SNP/ Genotype	Sex		Age at diagnosis		Disease activity		Disease extent			Food type		Social status	
	Male	Female	15–40	>40	Remission	Active	Rectum	Left colon	Pancolitis	Veg.	Non veg.	Rural	Urban
**D299G/T399I** (TLR4/ TLR4)													
AA/CC (Wild)	194	113	186	121	140	167	119	64	124	184	123	137	170
AG/CT (Het.)	13	08	16	05	16	05	03	06	12	14	07	11	10
Total	207	121	202	126	156	172	122	70	136	198	130	148	180
Major allele freq.	0.93	0.93	0.92	0.96	0.89	0.97	0.97	0.91	0.91	0.92	0.94	0.92	0.94
Minor allele freq.	0.06	0.06	0.07	0.03	0.10	0.02	0.02	0.08	0.08	0.07	0.05	0.07	0.05
**D299G/N592S** (TLR4/TLR5)													
AA/AA (Wild)	201	118	193	126	156	169	119	69	137	194	130	143	176
AG/AG (Het.)	06	03	09	00	06	03	03	01	04	04	05	05	04
Total	207	121	202	126	162	172	122	70	141	198	135	148	180
Major allele freq.	0.97	0.97	0.95	1.0	0.96	0.98	0.97	0.98	0.97	0.97	0.96	0.96	0.97
Minor allele freq.	0.02	0.02	0.04	0.0	0.03	0.01	0.02	0.01	0.02	0.02	0.03	0.03	0.02

Freq. = Frequency, Veg. = Vegetarian, Het. = Heterozygous mutant.

### Role of SNPs in Modulating Cytokine Expression

**Fig 2:**
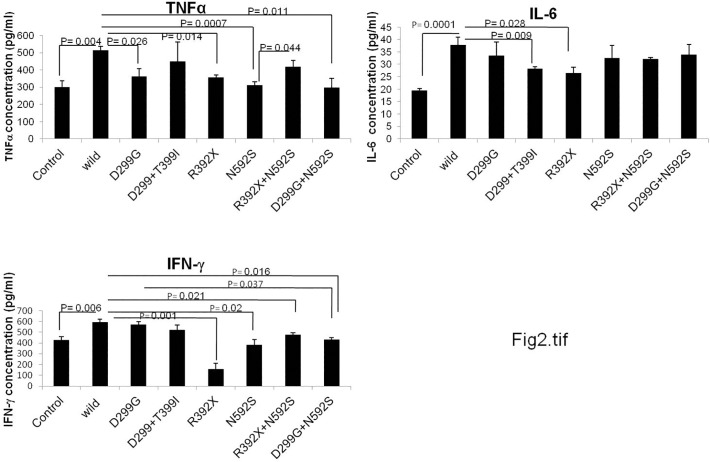
Analysis of cytokines level in UC patients and controls. TNFα, IL-6 and IFNγ levels were measured in human blood plasma samples (n = 4–8) by ELISA. Cytokines level were measured in wild type controls, and in wild type patients as well as with different genotype category of patient samples. The data is represented as mean value of 4–8 samples in each group. The significance level of α ≤0.05 was chosen for all sets. pg = picograms, ml = milliliter.

To check the role of polymorphism in cytokine homeostasis, we measured cytokine levels (TNFα, IL-6 and IFNγ) in control and UC patient samples (wild type and in individuals possessing single SNP or co-existing SNPs). All controls included for this analysis were non IBD and wild type (for all SNPs studied here). Level of TNFα (p = 0.004), IL-6 (p = 0.0001) and IFNγ (0.006) was significantly increased in wild type UC patients (wild type for all SNPs) as compared to controls (Fig. 2) suggesting that the change is due to the disease condition only. However, UC patients carrying D299G SNP in TLR4 gene revealed decreased level of TNFα (p = 0.026) as compared to wild type UC patients. Patients with R392X SNP in TLR5 gene also exhibited significantly decreased level of TNFα (p = 0.014), IL-6 (p = 0.028) and IFNγ (p = 0.001) as compared to wild type UC patients. In case of patients with N592S SNP (in TLR5 gene), TNFα (p = 0.0007) and IFNγ (p = 0.02) levels decreased significantly (Fig. 2). We were not able to measure cytokine level for T399I SNP due to insufficient number of samples carrying only this SNP.

Since during SNP analysis, we recorded few patients having multiple mutations either in two different loci of the TLR gene or two different SNPs located in different TLR gene, therefore it was tempting to measure the cytokine levels in patient samples carrying coexisting SNPs. Hence, we further analyzed three SNP interaction groups (D299G/T399I, R392X/N592S and D299G/N592S) that were found common in our study population group. There was decreased level of IFNγ (p = 0.021) in UC patients carrying R392X/N592S combinations compared to wild type UC patients. TNFα and IL-6 levels were also compromised as compared to wild type but did not attend a significant value. TNFα (p = 0.011) and IFNγ (p = 0.016) levels were significantly decreased in D299G/N592S combination compared to wild type patients. In samples with D299G/T399I combination, level of all the three cytokines decreased as compared to wild type but only IL-6 level attained a statistical significant value (p = 0.009) (Fig. 2).

## Discussion and Conclusions

We have reported here for the first time, the frequencies of R392X (4.4%) and N592S (9%) polymorphisms in TLR5 gene and their association with UC patients in North Indian population. Supporting our observation, a study from Jewish population [9] shows that R392X (TLR5-stop) have 6% and 0.9% hetrozygosity in UC and CD patients respectively as compared to unaffected relatives (5.4%) and unrelated controls (6.5%). Further, it was observed that in non Jewish population, this SNP was not associated with CD and UC. In R392X heterozygous CD patients, lower level of flagellin (the ligand of TLR5) was recorded using specific antibody [9]. Previous studies also conclude that SNP was associated with increased risk for urinary tract infection in adult women [17]. It can affect lung function in cystic fibrosis patients as well [18]. N592S SNP of TLR5 gene was first discovered in normal Caucasian population [19]. Recent study shows that in German population, N592S was associated with decreased survival of human colorectal cancer patients [20]. According to available literature, we are reporting here for the first time the association of this SNP in UC patients of North Indian population.

TLR2 and its co receptor TLR1 and TLR6 recognize and bind to lipoproteins, an important surface antigen of the Gram-negative bacteria [21–22]. Absence of any association of R753Q polymorphism in TLR2, R80T in TLR1and S249P in TLR6 gene with the disease in UC patients of our population supports earlier studies carried out in Korean population [23] and in Chinese population [24]. However, it was not in agreement with the earlier study, R80T and R753Q SNPs were found to be associated with pancolitis in UC patients of Belgian population [10]. Interestingly, we observed almost 100% mutation frequency for S249P SNP of TLR6 gene in our population however no association with the disease was observed. A negative association of the above SNP with UC was also observed in Belgian population [10]. SNP S258G in TLR3 gene was also not found to be associated with UC patients of North Indian population. Polymorphisms in the TLR3 gene was found to be associated with other diseases like type 1 diabetes and Stevens Johnson syndrome [25–26]. We have summarized the above available information in S1 Table.

Further we show that polymorphism combination in TLRs affects cytokine level in UC patients. Previously, Pierik et al, 2006 [10] demonstrated that polymorphism in TLR1, 2 and 6 contribute to more extensive disease phenotype in UC and CD. But this study was based only on genotype-phenotype analysis. Another study by Gazouli et al, 2005 [27] also shows that co-existence of mutations in TLR4, CD14 and NOD2 have phenotype with increase in disease severity. A recent study conducted in HT-29 cell line, shows that mRNA level of TNFα decreased in flagellin stimulated homozygous mutant cells (mutant for N592S, R392X and F616l SNP of TLR5 gene) as compared to TLR5 wild type cells [20]. In this study we observe that SNPs in TLR5 gene affects cytokines (TNFα, IL-6, IFNγ) level in UC, when present singly or in combination with TLR4 SNPs. Yang et al, 2012 also showed that T399I can modulate TNF alpha level in nasopharyngeal carcinoma [28]. Previous report on pulmonary lesion score in pigs showed that polymorphism in TLRs modulates cytokine level [29]. Since this study was in pigs and ours is in humans but it indicates role of SNPs in modulating the TLR pathway. But this is the first study in UC patients showing that different genotypes of TLR genes play an important role in modulating cytokine level as well as their phenotype.

TLR signaling and gut bacteria are important tool for regulation of intestinal immune system. Studies show that signaling to T cells (T-regulatory and T-effector cells) via TLRs can affect IBD pathogenesis to bacterial proteins for example flagellin, which is important for this signaling [7]. So it can be speculated that mutations in TLR genes can affect this signaling pathway. Our observation shows that patients having coexisting SNPs in TLR gene can modulate cytokine homeostasis. In support of our observation Nguyen et al, 2006 [30] indicated that there is a role of genetic variation in IBD pathogenesis. Study was conducted in non Hispanic white, non Hispanic African-American and in Hispanic IBD patients. In addition to the SNPs, racial differences also influence the disease manifestation like disease location, disease extension and extra intestinal manifestations.

To the best of our knowledge, this study has made a valuable contribution to draw a plausible link between TLR gene polymorphisms in single loci or multiple loci within or between two TLR genes and have shown how they modulate the expression of pro-inflammatory cytokines in the pathogenesis of UC. However, replication in additional UC patient cohorts, case control studies and further functional work to dissect out the role of TLR polymorphisms in vitro conditions, are clearly warranted.

## Supporting Information

S1 Fig Schematic representation showing location of TLR5 gene, SNPs, and genotyping. For scoring R392X SNP in the TLR5 gene, restriction digestion was carried out using Dde1 enzyme that generates one band of 346 bp in wild type, three bands of 346bp, 255 bp and 91 bp in heterozygous mutant and two bands of 255 + 91 bp in homozygous mutant. For the other SNP N592S in TLR5 gene, restriction digestion with Mun1 enzyme yielded two bands of 186+128bp in wild type. Heterozygous mutant exhibited three bands of 314bp, 186bp and 121bp and single band of 314bp in homozygous mutant.
(TIF)

S1 Table Worldwide association studies with different SNPs of TLR genes. (DOC)
