# Supplementary figures and images for: Association of TLR5 Gene Polymorphisms in Ulcerative Colitis Patients of North India and Their Role in Cytokine Homeostasis
Source: PLoS One. 2015 Mar 19;10(3):e0120697. doi: 10.1371/journal.pone.0120697 (PMC4366177; doi:10.1371/journal.pone.0120697)

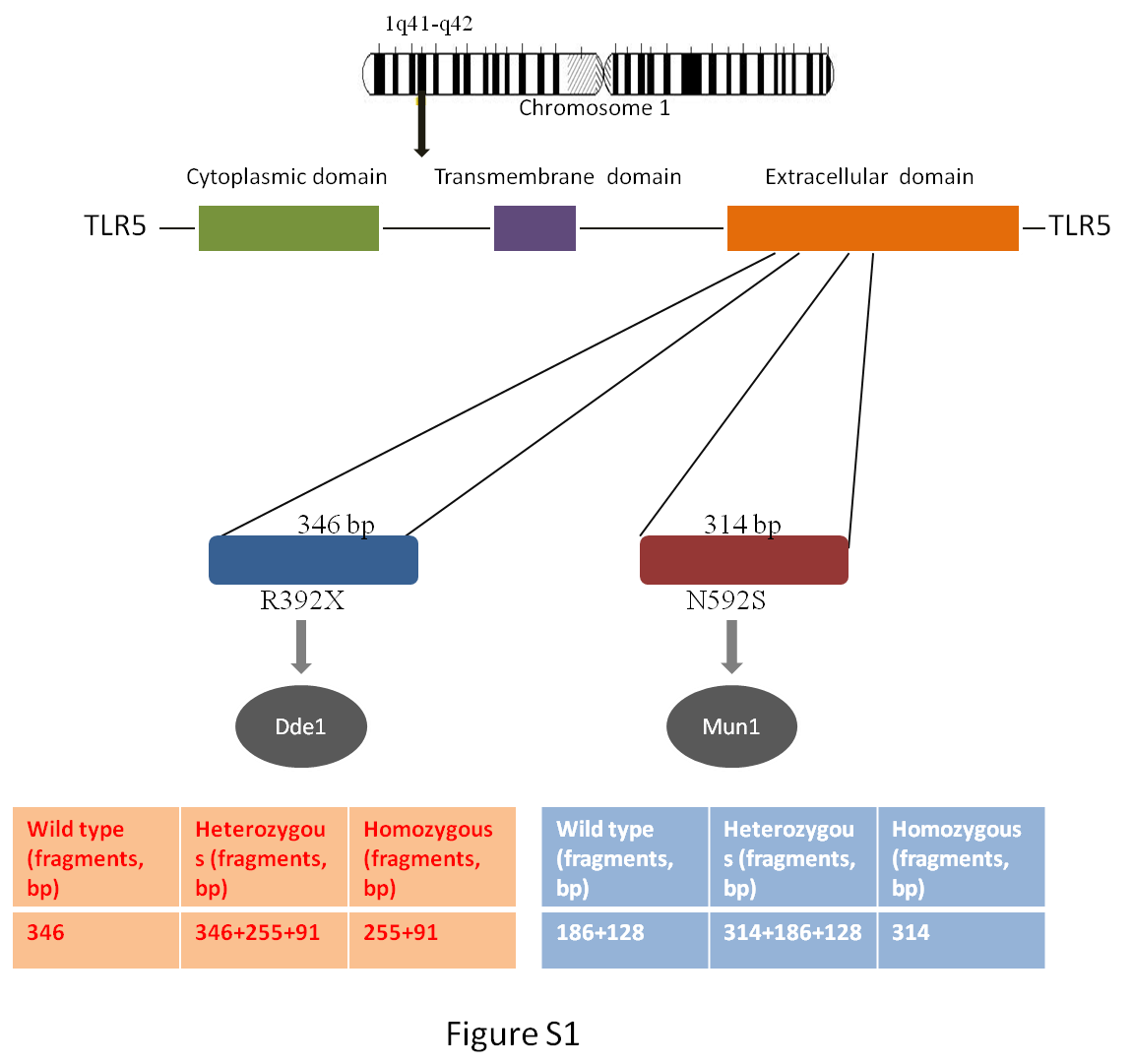

Supplement: S1 Fig — For scoring R392X SNP in the TLR5 gene, restriction digestion was carried out using Dde1 enzyme that generates one band of 346 bp in wild type, three bands of 346bp, 255 bp and 91 bp in heterozygous mutant and two bands of 255 + 91 bp in homozygous mutant. For the other SNP N592S in TLR5 gene, restriction digestion with Mun1 enzyme yielded two bands of 186+128bp in wild type. Heterozygous mutant exhibited three bands of 314bp, 186bp and 121bp and single band of 314bp in homozygous mutant. (TIF) [file pone.0120697.s001.tif]
