# Supplementary material for: Association of TLR5 Gene Polymorphisms in Ulcerative Colitis Patients of North India and Their Role in Cytokine Homeostasis
Source: PLoS One. 2015 Mar 19;10(3):e0120697. doi: 10.1371/journal.pone.0120697 (PMC4366177; doi:10.1371/journal.pone.0120697)
Supplement: S1 Table — (DOC) [file pone.0120697.s002.doc]

**S2 Table. Worldwide association studies with different SNPs of TLR genes (TLR 1,**

**TLR2, TLR3, TLR5 and TLR6)**

| Study | Population | Disease/Normal | Associated SNP |
| --- | --- | --- | --- |
| Gewirtz et al^9^ | Jewish | Inflammatory bowel disease | TLR5-R392X |
| Hawn et al^17^ | Caucasian | Urinary tract infection | TLR5-R392X |
| Haerynck et al^18^ | Belgium | cystic fibrosis | TLR5-R392X |
| Kao et al^19^ | Caucasian | Normal subjects | TLR5-N592S |
| Klimosch et al^20^ | German | Colorectal cancer | TLR5-N592S |
| Kim et al^23^ | Korean | Inflammatory bowel disease | TLR1-R80T,  TLR2-R753Q, TLR6-S249P |
| Chen et al^24^ | Zhuang population from China | Inflammatory bowel disease | TLR2-R753Q |
| Pierik et al^10^ | Belgian | Inflammatory bowel disease | TLR1-R80T, TLR2-R753Q, TLR6-S249P |
| Pirie et al ^25^ | Black South Africans | Type 1 diabetes | TLR3-S258G |
| Ueta et al^26^ | Japanese | Stevens Johnson syndrome | TLR3-S258G |
